# Incidence of Intraocular Pressure Elevation following Intravitreal Ranibizumab (Lucentis) for Age-related Macular Degeneration

**DOI:** 10.5005/jp-journals-10008-1213

**Published:** 2017-01-18

**Authors:** Gustavo MSM Reis, John Grigg, Brian Chua, Anne Lee, Ridia Lim, Ralph Higgins, Alessandra Martins, Ivan Goldberg, Colin I Clement

**Affiliations:** 1 Consultant, Glaucoma Unit, Sydney Eye Hospital, Sydney, New South Wales, Australia; 2 Associate Professor, Glaucoma Unit, Sydney Eye Hospital, Sydney; Discipline of Ophthalmology, Central Clinical School, University of Sydney Sydney, New South Wales, Australia; 3 Clinical Lecturer and Staff Specialist, Glaucoma Unit, Sydney Eye Hospital, Sydney; Discipline of Ophthalmology, Central Clinical School, University of Sydney Sydney, New South Wales, Australia; 4 Clinical Lecturer and Staff Specialist, Glaucoma Unit, Sydney Eye Hospital, Sydney; Discipline of Ophthalmology, Central Clinical School, University of Sydney Sydney, New South Wales, Australia; 5 Clinical Lecturer and Visiting Medical OfficerGlaucoma Unit, Sydney Eye Hospital, Sydney; Discipline of Ophthalmology, Central Clinical School, University of Sydney Sydney, New South Wales, Australia; 6 Visiting Medical Officer, Glaucoma Unit, Sydney Eye Hospital, Sydney; Discipline of Ophthalmology, Central Clinical School, University of Sydney Sydney, New South Wales, Australia; 7 Consultant, Department of Ophthalmology, Moorfields Eye Hospital London, United Kingdom; 8 Clinical Associate Professor, Glaucoma Unit, Sydney Eye Hospital, Sydney; Discipline of Ophthalmology, Central Clinical School, University of Sydney Sydney, New South Wales, Australia; 9 Clinical Lecturer and Staff Specialist, Glaucoma Unit, Sydney Eye Hospital, Sydney; Discipline of Ophthalmology, Central Clinical School, University of Sydney Sydney, New South Wales, Australia

**Keywords:** Age-related macular degeneration, Intraocular pressure, Ranibizumab.

## Abstract

**Aim:**

The aim of this article is to evaluate the rate of patients developing sustained elevated intraocular pressure (IOP) after ranibizumab (Lucentis) intravitreal (IVT) injections.

**Design:**

This is a retrospective study.

**Participants:**

Charts of 192 consecutive patients receiving Lucentis for age-related macular degeneration (AMD) were retrospectively reviewed.

**Materials and methods:**

We enrolled patients with at least two IOP measurements between injections. Elevated IOP was defined as >21 mm Hg with an increase of at least 20% from baseline. Noninjected contralateral eyes of the same patient cohort were used as control.

**Main outcome measures:**

Primary outcome was defined as elevated IOP. Secondary outcomes were presence and type of glaucoma, number of injections, and time to IOP elevation.

**Results:**

Elevated IOP occurred at a significantly higher rate in eyes receiving IVT ranibizumab (7.47%; n = 9) compared with control (0.93%; n = 1). Patients with preexisting glaucoma or ocular hypertension (OHT) were more likely to develop elevated IOP after IVT ranibizumab injection.

**Conclusion:**

Intravitreal ranibizumab injections are associated with sustained IOP elevation in some eyes.

**How to cite this article:**

Reis GMSM, Grigg J, Chua B, Lee A, Lim R, Higgins R, Martins A, Goldberg I, Clement CI. The Incidence of Intraocular Pressure Elevation following Intravitreal Ranibizumab (Lucentis) for Age-related Macular Degeneration. J Curr Glaucoma Pract 2017;11(1):3-7.

## INTRODUCTION

According to the World Health Organization (WHO), age-related macular degeneration (AMD) is the primary cause of irreversible blindness in developed countries among people aged over 50, and the third leading cause of blindness worldwide.^1^ In the majority of patients with AMD, vision loss is due to the development of choroidal neovascularization (CNV), which is characterized by aberrant growth of new blood vessels from the choroid into or underneath the retina. These abnormal vessels leak blood and/ or fluid causing retinal damage by edema with cystic degeneration and fibrous scarring.

Ranibizumab is an antivascular endothelial growth factor (anti-VEGF) agent that was approved by the Food and Drug Administration in June 2006 for the treatment of CNV due to AMD.^2^ It is a recombinant humanized immunoglobulin monoclonal antibody fragment (Fab) directed against the receptor-binding domain of all active isoforms of VEGF-A inhibiting cell proliferation (angio-genesis) and reducing vascular leakage.^3^ It is initially used as a 0.05 mL (0.5 mg) intravitreal (IVT) injection every 4 weeks, with frequency thereafter tailored according to the clinical response.

The most common adverse ocular reactions reported in patients receiving ranibizumab included intraocular inflammation, cataract, vitreous hemorrhage, and increased intraocular pressure (IOP).^3^

It is known that an acute volume-related ocular hypertension (OHT) occurs transiently after IVT injections; however, within 30 to 60 minutes, the ocular pressure usually returns to baseline.^4^ Sustained OHT after anti-VEGF injections has been reported in both case series^2,5^ and retrospective studies^6,7^; the incidence ranges from 3 to 9%.

Our study aims to establish the risk of sustained elevated IOP after ranibizumab injections in an Australian population in order to assess the safety profile of such treatment and whether presence of glaucoma or number of injections could be identified as a risk factor for IOP increase.

## MATERIALS AND METHODS

The medical records of consecutive patients receiving IVT ranibizumab (Lucentis, Genetech, San Francisco, CA, USA) for AMD at Sydney Eye Hospital were retrospectively reviewed. The study was approved by the South Eastern Sydney Local Health District Human Research Ethics Committee and conformed to tenants of the Declaration of Helsinki on Human Research.

We aimed to compare the highest recorded IOP after commencing IVT ranibizumab treatment with baseline IOP. Eyes were included for analysis if IOP, measured with Goldman tonometry, was clearly documented before commencing ranibizumab treatment and at all follow-up visits. As the contralateral noninjected eye was used as control, patients receiving bilateral treatment were excluded. Eyes with a history of IVT anti-VEGF treatment at another institution were also excluded. Demographic data, IOP, visual acuity, history of glaucoma or OHT, number of ranibizumab injections, time from onset of ranibizumab treatment, and IOP-reducing interventions were recorded. The IOP was considered elevated if at follow-up, a minimum 4 weeks following injection, measurement was >21 mm Hg with an increase of >20% from baseline on two consecutive visits.

All IVT injections were given using the same technique in an outpatient setting. In short, eyelids were cleaned with povidone-iodine after topical anesthesia with amethocaine hydrochloride 1% drops. A small bolus of subconjunctival lidocaine 1% was given immediately before ranibizumab (0.5 mg in 0.05 mL) was injected using a 30-gauge needle into the vitreous via either inferior or superior approach, 3.5 mm (pseudophakic eyes) or 4 mm (phakic eyes) from the corneoscleral limbus. Gentle local pressure with a cotton tip was applied for 10 seconds to avoid reflux, and chloramphenicol drops qid were routinely prescribed for a week following injection.

### Statistics

Categorical and continuous data were compared using Chi-squared and Student t-test respectively, in Numbers (v 3.6.1). The effect of age, gender, preexisting glaucoma or OHT, and number of injections on final IOP was analyzed using linear regression modeling (Wizard for Mac, v 1.7.20). A p-value <0.05 was considered significant.

## RESULTS

The medical records of 192 patients were reviewed. From these, 107 patients met the inclusion criteria. Baseline characteristics are presented in Tables 1 and 2.

Baseline acuity was significantly worse in control eyes compared with injected eyes (Table 2). This was, in part, due to the presence of end-stage AMD for which no IVT treatment was received. There was no significant difference in baseline IOP between injected and control eyes (Table 2). However, eyes receiving injections showed a statistically higher IOP compared with control eyes (17.13 *vs* 15.38 mm Hg; p = 0.02) at final follow-up.

Significantly more injected eyes displayed elevated IOP (>21 mm Hg and >20% from baseline) compared with controls (7.47 *vs* 0.93%; p = 0.03). In those eyes with IOP elevation, the mean highest IOP was 39.5 mm Hg. The number of injections given to eyes with elevated IOP was greater (19.43; 8-39) than eyes in which IOP did not rise (14.47; 2-62); however, there was considerable overlap and the difference was not significant.

Preexisting glaucoma or OHT was identified in 15 of 107 eyes (14.0%) receiving ranibizumab [primary open angle glaucoma, n = 7; OHT n = 5; pseudoexfoliative syndrome (PXF), n = 3). The IOP elevation occurred in 4 of 15 (26.7%) glaucoma eyes and 4 of 92 (4.3%) nonglaucoma eyes following injection. The rate of IOP elevation in eyes with preexisting PXF was very high (100%), although the overall number was small (n = 3).

Linear regression modeling revealed that age, baseline IOP, and preexisting glaucoma/OHT were significantly associated with highest recorded postinjection IOP (Table 3).

**Table 1:** **Table 1:** Baseline characteristics

*Number*			*107*	
Age (years)			76.87	
Male gender (%)			52.3	
Ethnicity (%)				
Caucasian			68.2	
Asian			19.6	
Arabic			7.5	
Other			4.7	

**Table 2:** **Table 2:** Acuity and IOP in injected and control eyes

		*Injected*		*Control*		*p-value*	
LogMAR acuity		0.69 ± 0.09		0.93 ± 0.18		>0.01	
Total number of		1,583		–		–	
injections (n)							
Mean number of		14.47 ± 1.84		–		–	
injections (n)							
Preinjection IOP (mm Hg)		15.26 ± 0.61		15.32 ± 0.57		0.45	
Postinjection IOP (mm Hg)		17.13 ± 1.51		15.38 ± 0.71		0.02	
Eyes with elevated IOP (%)		7.47		0.93		0.03	

LogMAR: Logarithm of the minimum angle of resolution

**Table 3:** **Table 3:** Linear regression modeling of variables influencing final IOP following intravitreal ranibizumab injection

*Variable*		*Coefficient*		*Standard error*		*p-value*	
Age		0.063		0.029		0.031	
Gender (male)		–0.242		0.641		0.707	
Baseline IOP		0.384		0.098		<0.001	
Number of injections		0.045		0.033		0.176	
Glaucoma/OHT		–2.44		0.873		0.007	

## CASE REPORTS

A summary of each case in which elevated IOP was documented following IVT ranibizumab follows.

### Case 1

A 79-year-old phakic female was using latanoprost and brimonidine/timolol fixed combination daily in both eyes for PXF glaucoma and underwent left selective laser trabeculoplasty (SLT) in 2009, 1 year prior to commencing ranibizumab for AMD in April 2010. Pretreatment IOPs were 18/16 mm Hg with 0.7 and 0.9 cup/disk ratio. After three injections over 3 months, IOP was 18/36 on the same treatment. Oral acetazolamide 250 mg three times daily (TDS) was given with left IOP reducing to 20 mm Hg. Systemic anhydrase inhibitor was tapered and a month later she had another pressure spike - 27/36 mm Hg. Oral medication (250 mg BD) was recommenced, with IOP improving to 18/32 mm Hg. Left trabeculectomy was carried out. Six months following surgery, IOP was 22/6 mm Hg, and the patient was still receiving ranibizumab injections.

### Case 2

A 78-year-old pseudophakic female with previous history of OHT commenced right eye ranibizumab in 2010. Pretreatment IOP was 19/18 mm Hg with latanoprost in both eyes. Following eight injections over 12 months, IOP was elevated to 62/14. Oral acetazolamide 250 mg TDS, apraclonidine TDS, and timolol maleate 0.5% BD were started, reducing IOP on the following day to 11/10 mm Hg. All medications except latanoprost were withdrawn. Two months later, following the ninth injection, IOP was 50/20 mm Hg. This improved to 17/10 with oral acet-azolamide 250 mg TDS and brimonidine/timolol fixed combination BD. After 2 weeks, SLT was performed. A month later, IOP was 18/17 on bimatoprost, brimonidine/ timolol fixed combination, and dorzolmaide BD. She continues to have IVT ranibizumab as needed, and IOP remains controlled.

### Case 3

A 75-year-old pseudophakic male with no known history of glaucoma commenced ranibizumab in 2010 in the Right eye (RE) with baseline IOP of 16/16 mm Hg. Following eight injections over 10 months, IOP increased to 54/17 mm Hg. He was given acetazolamide 250 mg TDS, timolol maleate 0.5% BD, and iopidine TDS following which IOP reduced to 22 mm Hg. Oral medication was discontinued and ranibizumab resumed 3 weeks later. At review, IOP was 44/17 mm Hg requiring treatment with bimatoprost and brimonidine/timolol fixed combination BD with IOP coming down to 24/16 mm Hg. Another ranibizumab was given 2 months later, and the next week, the IOP was 32/19 mm Hg. He underwent SLT with IOP improving to 18/12 within 2 weeks. However, another ranibizumab injection 3 months after SLT was followed by and resulting in an IOP of 8/17 on no medication 6 months after surgery. Six further ranibizumab injections have been tolerated during that period with no change to the IOP.

### Case 4

A 67-year-old phakic male with no history of glaucoma commenced left ranibizumab in 2009. The IOP increased from a baseline of 14/14 to 21/36 mm Hg after six injections over 7 months. With oral acetazolamide 500 mg, iopidine TDS, and pilocarpine TDS, IOP improved to 16/23 mm Hg. It remained controlled over the 18 months during which he had a further nine ranibizumab injections. However, after his 15th injection, IOP was elevated to 17/58 mm Hg. Back on maximum medical therapy, IOP only marginally improved to 47 mm Hg. The next day, he underwent a left combined phacoemulsification and viscocanalostomy. Three months after surgery, IOP was 18/18 mm Hg without medication. Three further ranibizumab injections have been given during this time.

### Case 5

A 65-year-old pseudophakic male with no history of glaucoma commenced ranibizumab in February 2011 to the right eye for AMD. Past ocular history included a blind left eye secondary to chronic retinal detachment, high myopia, and right retinal tears treated with cryo-therapy. Baseline IOP was 11/12 mm Hg, but increased to 32/14 mm Hg after six injections. Right IOP improved to 21 mm Hg on latanoprost. He had a further six injections over 11 months after which IOP increased to 40 mm Hg in the right eye. Oral acetazolamide 250 mg TDS and brimonidine/timolol fixed combination BD was started and IOP remained at 10/10 mm Hg on topical hypotensive medication only 6 months later.

### Case 6

A 67-year-old pseudophakic male with advanced PXF glaucoma started on ranibizumab treatment in 2008 had a baseline IOP at 16/16 mm Hg with travoprost/ timolol fixed combination daily and dorzolamide BD. Previous cyclodiode laser and tube implant surgery were performed years before commencing on ranibizumab. After 7 months of IVT injections, the IOP started to rise to 25/18 mm Hg and g. brimonidine was added on his RE. Pressure was well controlled since then when it rose to 26/17 mm Hg 3 months later requiring systemic acetazolamide 250 mg QID. It was then tapered to TDS, and the patient had a new IOP spike: 37/18 mm Hg. Cyclodiode laser was performed achieving good IOP control. The patient had another four injections between surgery and last follow-up, where IOP was 10/15 mm Hg on bimatoprost/timolol fixed combination daily and brimonidine BD.

### Case 7

An 84-year-old pseudophakic female with PXF glaucoma had baseline IOP of 15/12 on latanoprost. Ranibizumab injections were commenced for AMD in her RE in 2008. She had IOP ranging from 15 to 24 mm Hg within the next 4 years of follow-up, receiving a total of 36 injections during this period. However, on a routine visit, her IOP was 46/15 mm Hg without any symptoms. Oral acetazolamide 250 mg was started with topical brimo-nidine/timolol fixed combination as well as latanoprost. However, since she was unresponsive to medical treatment, an anterior chamber paracentesis was carried out to reduce the IOP to 12 mm Hg. She was sent home with acetazolamide 125 mg BD, g. latanoprost, and g. brimonidine/timolol. A week later, pressure levels were 11/10 mm Hg, and oral acetazolamide was replaced by brinzolamide. When assessed again, IOP was 42/14 mm Hg and oral acetazolamide was once again prescribed with SLT performed 180°. Three months after the SLT, IOP on medical therapy was 26/14 mm Hg. Due to her poor visual acuity (hand movements in both eyes) and advanced age, the patient’s relatives declined surgical intervention.

### Case 8

An 81-year-old pseudophakic female with no prior history of glaucoma commenced ranibizumab in 2010 to the right eye for AMD. Baseline IOP was 18/19 mm Hg on no treatment. Following five IVT injections of ranibizumab to the right eye over an 8-month period, the IOP rose to 27/20 mm Hg. After commencing latanoprost to the right eye, the IOP fell to 21 on that side. The patient remained on latanoprost and continued to have IVT ranibizumab to the right eye as required. No further significant elevations in IOP were recorded.

## DISCUSSION

The aim of this study was to assess the rate of sustained IOP elevation in eyes receiving IVT ranibizumab for AMD. Using IOP > 21 mm Hg and IOP > 20% from baseline as the definition of elevated IOP, this study has shown sustained IOP elevation in 7.47% of eyes receiving IVT ranibizumab. Preexisting glaucoma or OHT, higher baseline IOP, and increasing age were risk factors for sustained IOP elevation in this series.

Our study results are consistent with the findings of a number of other studies. Using criteria of IOP of at least 22 mm Hg and elevation of at least 6 mm Hg at two consecutive visits, Bressler et al^8^ reported IOP elevation in 9.5% of participants receiving ranibizumab *vs* 3.4% receiving a sham injection. This is despite excluding eyes with preexisting glaucoma, a design that may result in an underestimation of the anti-VEGF effect. Another significant study by Freund et al,^9^ in which data from the VEGF Trap-Eye study and VIEW 1 and 2 studies were pooled, compared the effects of IVT aflibercept and ranibizumab. They report a higher rate of IOP > 21 mm Hg in eyes receiving 4-weekly ranibizumab compared with eyes receiving one of three aflibercept regimens (20.2 *vs* 14.2%, 12.1%, or 12.5%).

Smaller studies like our own have reported similar findings, providing further evidence of an association. Adelman et al^7^ identified 4 eyes out of 116 (3.4%) treated for AMD with either bevacizumab or ranibizumab injections that developed sustained OHT after a mean of 13 treatments. Good et al^6^ found sustained IOP elevation in 13 of 215 eyes (6%) after a median of nine injections of ranibizumab and/or bevacizumab, noting a higher prevalence in eyes receiving only bevacizumab (9.9%) compared with those receiving only ranibizumab (3.1%). They further noted higher rates in patients with preexisting glaucoma (33 *vs* 3.1% in eyes without preexisting glaucoma). Agard et al^10^ found that 10 out of 217 eyes (4.6%) experienced IOP elevation above 25 mm Hg after a mean 6.7 IVT injections of an anti-VEGF agent.

The mechanism by which sustained IOP elevation occurs following IVT ranibizumab treatment is unknown. Currently three mechanisms seem likely; these may act in isolation or have a combined effect. The first is mic-roparticle obstruction, a process involving diffusion of the 48-kDa ranibizumab antibody fragment into the anterior chamber leading to physical obstruction and increased resistance within the trabecular meshwork. A similar process may occur due to the presence of silicone droplets or protein microaggregates from delivery equipment or packaging. Low-grade inflammation following injection is considered another potential cause, as it has the potential to alter fibroblast proliferation and scar deposition within the trabecular meshwork. Repeat trauma to the trabecular meshwork due to immediate and recurrent IOP spikes following injection has also been proposed.

The above-proposed mechanisms increase outflow resistance via changes to the trabecular meshwork. This is consistent with the finding of this study and others that glaucoma or OHT is a risk factor for sustained IOP elevation following IVT ranibizumab. It is likely eyes with glaucoma or OHT have reduced outflow facility compared with normotensive nonglaucomatous eyes. Further injury to trabecular meshwork from micropar-ticle obstruction, inflammation, or spiking IOP at the time of injection may compromise outflow further to the point that an IOP within the normal range cannot be maintained.

Other risk factors for IVT ranibizumab have been reported and include number of injections, use of corti-costeroids, and phakic eyes.^11^ Again, these associations may be explained on the basis of effect on outflow facility. Number of injections could relate to IOP spiking with incremental increase in trabecular meshwork damage. Corticosteroids are well known to cause trabecular meshwork extracellular matrix remodeling. Phakic eyes have higher IOP than pseudophakic eyes. The mechanism for this observation is unknown, but changes to outflow facility have been implicated. Our finding that preexisting glaucoma or OHT, increasing age, and higher baseline IOP were positive predictors of developing sustained IOP is consistent with the theory that this response is mediated via an effect on outflow facility.

In this study, we used the contralateral noninjected eye as control. With this design, there is potential for the control eye to be exposed to ranibizumab via systemic absorption, as has been demonstrated in some animal models. For example, bilateral simultaneous IVT injection in monkeys of 0.5 mg ranibizumab is associated with ranibizumab antibody detection in serum peaking 3 hours after injection.^12^ However, a study in rabbits of monocular injections was not associated with serum ranibizumab antibody detection.^13^ These data suggest there is potential for systemic absorption, but the likelihood with monocular injection is uncertain. A recent meta-analysis suggests no increased risk of systemic cardiovascular events related to either ranibizumab or bevacizumab,^14^ implying that if systemic absorption occurs, its effect is minimal. That we observed a significant difference in the rate of IOP elevation between injected and control eyes is consistent with this. If anything, the control design in this study would serve to underestimate the effect on IOP. Interestingly, the only control eye that displayed elevated IOP in this study was in a patient who also displayed elevation in the injected eye.

In conclusion, this study found that sustained elevated IOP occurred in 7.47% of eyes receiving IVT ranibizumab for AMD. Risk factors for this finding included preexisting glaucoma/OHT, baseline IOP, and increasing age. This finding is consistent with similar studies and suggests repeat IVT ranibizumab has an effect on outflow facility in some eyes. The results highlight the importance of monitoring IOP in eyes receiving IVT ranibizumab.
